# Shape, Size and Bilateral Asymmetry of the Humerus and Femur in the Common Swift (*Apus apus*)

**DOI:** 10.3390/ani15233401

**Published:** 2025-11-25

**Authors:** Eylem Bektaş Bilgiç, Edyta Pasicka, Aycan Korkmazcan, Nicoleta Manuta, Buket Çakar, Ebuderda Günay, Gökhan Gün, Ozan Gündemir

**Affiliations:** 1 Department of Surgery, Faculty of Veterinary Medicine, Istanbul University-Cerrahpasa, Istanbul 34320, Türkiye; eylem.bilgic@iuc.edu.tr; 2 Department of Biostructure and Animal Physiology, Faculty of Veterinary Medicine, Wrocław University of Environmental and Life Sciences, 50-375 Wroclaw, Poland; 3 Institute of Graduate Studies, Istanbul University-Cerrahpasa, Istanbul 34320, Türkiye; aycan.korkmazcan@ogr.iuc.edu.tr (A.K.); nicoletamanu-ta@ogr.iuc.edu.tr (N.M.); buket.cakar@ogr.iuc.edu.tr (B.Ç.); 4 Department of Wild Animal Disease and Ecology, Faculty of Veterinary Medicine, Istanbul University-Cerrahpasa, Istanbul 34320, Türkiye; ebuderda.gunay@iuc.edu.tr; 5 Department of Molecular Biology and Genetics, Bogazici University, Istanbul 34342, Türkiye; gokhan.gun@bogazici.edu.tr; 6 Department of Anatomy, Faculty of Veterinary Medicine, Istanbul University-Cerrahpasa, Istanbul 34320, Türkiye

**Keywords:** avian anatomy, geometric morphometrics, bilateral shape variation, avian flight adaptation, limb skeletal loading, Procrustes analysis, aerial specialists

## Abstract

Common swifts are extreme aerial specialists that spend most of their lives in flight and use their legs mainly for clinging rather than locomotion. Because functional load is therefore expected to be concentrated on the wing, we hypothesized that the forelimb would exhibit stronger left–right differentiation than the hindlimb. In addition to testing this hypothesis, we used landmark-based geometric morphometric methods to describe humeral and femoral shape in common swifts and to test the effects of sex and body size on bone morphology. The humerus showed clear directional asymmetry: the effect of side explained 13.8% of total shape variance (F = 42.0, *p* = 0.001), indicating a consistent left–right shift across individuals. The femur also exhibited significant but weaker directional asymmetry, with side accounting for 5.4% of variance (F = 19.3, *p* = 0.001). In both bones, the individual term explained the largest proportion of variation, whereas residual variance (containing fluctuating asymmetry and measurement error) was moderate (≈27% in the humerus, ≈23% in the femur). Allometric regressions showed a weak but significant size–shape relationship for the humerus and only a marginal effect for the femur, and males and females showed almost complete overlap in the distribution of humeral and femoral shapes. Sex had no detectable effect on humeral or femoral shape or asymmetry, and body size explained only a modest proportion of shape variation in both elements. Overall, our results support the functional expectation: the more intensively used forelimb element is also the more directionally asymmetric one, whereas the femur of this largely aerial bird remains comparatively more symmetrical.

## Simple Summary

The common swift is a bird that spends almost its entire life in the air. It uses its wings all the time for flying but uses its legs mostly just to cling to walls or nesting places. Because the wings work much harder than the legs, we wanted to see whether the wing bone shows a clearer difference between the left and right sides than the leg bone. We compared the shapes of the upper wing bone (the humerus) and the upper leg bone (the femur) from the same birds. The wing bone showed a clear and consistent difference between left and right, while the leg bone showed a weaker difference. This means that the part of the body that is used more in daily life also shows a stronger trace of this use in its shape. Knowing this helps us understand how daily behavior, such as almost constant flight, can leave a mark on the skeleton of very aerial bird species.

## 1. Introduction

The common swift (*Apus apus*) is one of the most aerial of birds, spending virtually its entire life in flight [1,2]. It breeds throughout temperate Eurasia (Europe to Asia) on vertical cliffs and in buildings and then migrates each year to sub-Saharan Africa for winter, covering hundreds of kilometers per day [2,3]. Swifts exhibit extreme specializations for flight: they have very long, narrow, scythe-shaped wings and a streamlined body, yielding very high wing aspect ratios and wing loading [4,5]. These adaptations enable remarkable performance, for example, *Apus apus* has been clocked flying at up to ~31.1 m/s (≈112 km/h) and routinely covering 250+ km in a single day [6,7,8]. Aside from their wings, swifts possess a unique “grasping” foot (with all four toes fused forward) that lets them cling to vertical nesting sites; otherwise, they remain aloft, catching aerial insects in flight (forming them into throat boluses as they glide) [9]. These traits, life spent almost entirely on the wing, insectivorous feeding on the wing, and specialized wing morphology, underscore the common swift’s exceptional flight-focused ecology.

Asymmetry in wing shape can arise from developmental perturbations but may also be constrained by function [10,11,12]. Fluctuating asymmetry (FA), the small random left–right differences caused by imprecision during development, can increase when a bird experiences genetic or environmental stress [13,14]. For example, factors such as parasite load, food shortage or toxins during feather growth can elevate FA, making it an indicator of developmental instability or individual quality [15,16]. Conversely, flight performance imposes strong functional selection for symmetry: even slight asymmetries can reduce aerodynamic efficiency. Indeed, theoretical and experimental studies suggest that symmetry may improve aspects of flight performance. For example, Swaddle and Witter [16] found that starlings forced to fly in cluttered environments grew significantly more symmetric wing feathers and achieved better flight performance. Beyond feathers and external wing shape, bilateral asymmetry has also been documented at the level of avian limb bones. For example, fluctuating asymmetry in leg bone traits has been used as an indicator of bone quality and developmental stress in broiler chickens [17], and skeletal asymmetry of limb bones has been linked to footedness in parrots [18].

The aim of this study is to compare bilateral shape symmetry between the two main components of the locomotor system in the common swifts (*Apus apus*), which are highly specialized for powered flight: the forelimb (humerus) and the hindlimb (femur). Because this species spends most of its life in the air and performs feeding as well as orientation are carried out during flight whereas landing and terrestrial locomotion are very limited, the main functional load is expected to fall on the wing bones. Based on this functional asymmetry, we hypothesized that the forelimb element actively used in flight (the humerus) would exhibit a higher degree of left–right asymmetry, whereas the hindlimb element (the femur), which is used mainly for clinging and limited support, would be comparatively more symmetrical. In addition to this primary hypothesis, the study also aimed to describe the overall pattern of shape variation in both bones, visualizing which regions of the humerus and femur contributed most to shape change and how these changes were distributed between sexes. Furthermore, to test the relationship between size and shape, Procrustes shape coordinates were regressed on log-transformed centroid size, allowing us to evaluate how much of the observed shape differences could be explained by allometry (shape change associated with increasing body size).

## 2. Materials and Methods

### 2.1. Samples

This study was conducted on adult common swifts (*Apus apus*) that were submitted to the Istanbul University–Cerrahpaşa, Faculty of Veterinary Medicine, Department of Wildlife Diseases and Ecology between 2020 and 2024 and had died from natural or non-infectious causes. After excluding individuals with missing sex information, removing one specimen whose humerus was severely damaged during the cleaning process (which also prevented reliable sampling of the corresponding hindlimb bones), and excluding one clear shape outlier that showed extremely divergent variation and formed an isolated point in the exploratory PCA, the final dataset consisted of 82 specimens (50 females, 32 males). All specimens were fully ossified and considered adult at the time of death; therefore, no juvenile or subadult individuals were included in the analyses.

### 2.2. Molecular Determination of Sex in Apus apus

Sex was identified molecularly from feather material. Genomic DNA was isolated from 1 to 2 mm pieces of the feather calamus using a commercial tissue DNA kit (Genomic DNA from Tissue Kit, Macherey-Nagel GmbH & Co. KG, Dueren, Germany) according to the manufacturer’s protocol. To discriminate males and females, the conserved avian sex-linked CHD-Z and CHD-W loci were amplified with the widely used primer pair 2550F/2718R [19]. Each PCR was set up in a 25 µL volume containing 1× Taq buffer (Thermo Fisher Scientific, Waltham, MA, USA), 1 mM MgCl_2_, 0.2 mM of each dNTP, 0.5 µM of each primer, 0.5 U Taq DNA polymerase (Thermo Fisher Scientific), and approximately 1–5 ng of template DNA. Reactions were run on a t100 Biorad thermal cycler under the following thermocycling conditions: initial denaturation at 95 °C for 3 min; 40 cycles of 95 °C for 30 s, 50 °C for 30 s, and 68 °C for 45 s; and a final extension at 68 °C for 5 min. Amplified fragments were resolved on 2% agarose gels in TAE buffer at 100 V for 45 min, allowing visualization and differentiation of the CHD-W and CHD-Z bands.

### 2.3. Preparation of Bones

The humeri and femora were first macerated in warm water to soften adhering soft tissues and to allow careful separation of muscles without damaging the bone surface. Because the elements were relatively small and slender, tissue removal was performed manually with fine instruments rather than prolonged boiling. After gross cleaning, the bones were immersed in a 25% hydrogen peroxide solution for approximately 10 min to remove residual connective tissue and to gently lighten the surface. Immersion time was deliberately kept short to avoid any potential alteration of bone morphology, and specimens were visually inspected before and after treatment for signs of surface etching or deformation. Finally, all elements were rinsed with distilled water and air-dried at room temperature for several days in a well-ventilated environment before landmark digitization.

### 2.4. Digital Image Acquisition

All humerus and femur specimens were photographed prior to landmark digitization. Images were taken with a Canon EOS 500D digital camera (Canon, Tokyo, Japan) mounted on a fixed stand to ensure constant shooting geometry. Each bone was positioned on a flat, contrasting background and photographed from the same orientation and at a standardized distance of 24 cm from the camera lens. The camera settings and lighting were kept constant throughout the session to minimize distortion and shadow effects. All images were saved in PNG format at high resolution, and only photographs that were captured from the correct angle and distance were used for subsequent 2D landmarking.

### 2.5. Landmark Digitization

Two-dimensional landmark datasets were prepared from the standardized photographs of the humerus and femur. TPS files were first created using tpsUtil (version 1.74), with separate files opened for each bone and side (left and right) to keep the specimens organized [20]. The images were then imported into tpsDig2 (version 2.32), and fixed anatomical landmarks were digitized on each photograph according to the configuration defined for this study: 11 landmarks for the humerus and 12 landmarks for the femur [21]. All points were placed manually on clearly identifiable osteological features (proximal articular surface, shaft inflection points, and distal articular margins), and care was taken to digitize the right and left elements from the same view and scale. The completed TPS files, containing the final landmark coordinates, were saved and used directly in the subsequent geometric morphometric analyses in RStudio (version 2024.09.1) [22,23].

### 2.6. Statistical Analysis

All statistical procedures were carried out in R software (version 4.4.2), primarily using the geomorph package (v.4.0.9) [24]. Landmark coordinates for the humerus (11 landmarks) and femur (12 landmarks) were first imported from TPS files and subjected to a Generalized Procrustes Analysis to remove the effects of translation, rotation, and scale; this step produced Procrustes-aligned shape coordinates and centroid size (CS) for each specimen. To characterize the main axes of shape variation, separate principal component analyses (PCA) were then performed for the humerus and the femur. The distribution of specimens in PC1–PC2 space was plotted and color-coded by sex in order to visually assess whether males and females occupied different regions of morphospace, and deformation grids at the extremes of the PCs were inspected to interpret which anatomical regions contributed most to shape change.

Sex-related differences in both size and shape were evaluated using Procrustes ANOVA based on 999 permutations. For each bone, Procrustes shape coordinates were tested against sex to determine whether a significant proportion of the variation could be attributed to sexual dimorphism, and centroid size was likewise compared between females and males. Bilateral asymmetry was assessed on the subset of individuals for which both left and right elements were available. Right-side bones were mirrored to match the orientation of the left side, aligned together, and analyzed with a Procrustes-based model in which the factor “side” represented directional asymmetry (DA), whereas the factor “individual” captured among-individual variation. Because each side was digitized only once, the unexplained residual component was interpreted as fluctuating asymmetry (FA) combined with measurement error. In addition, the relationship between size and shape was examined by regressing Procrustes coordinates on log-transformed centroid size to test for the presence of allometry, allowing us to distinguish shape differences associated with body/element size from genuine asymmetry. For all permutation-based tests, the significance level was set at α = 0.05, and results were reported as sums of squares, proportion of explained variance (R^2^), F values, and permutation-derived *p* values.

## 3. Results

### 3.1. Size and Shape

Sex had no detectable effect on size or shape in the sampled material. For the left humerus, females had an estimated centroid size of 703.95 units, while males were only slightly larger (703.95 + 0.75 = 704.70 units), and this difference was statistically non-significant (F_1_,_80_ = 0.019, *p* = 0.892). For the left femur, the pattern was the same but in the opposite direction: females averaged 1062.99 units, whereas males were very slightly smaller (1062.99 − 1.54 = 1061.46 units), again with no statistical support for a sex effect (F_1_,_80_ = 0.054, *p* = 0.816). Shape analyses using Procrustes ANOVA likewise showed that sex explained only about 1% of the variation in humeral shape (R^2^ = 0.009, F = 0.75, *p* = 0.694) and femoral shape (R^2^ = 0.009, F = 0.75, *p* = 0.623). Taken together, these results indicate that, in this *Apus apus* sample, neither the forelimb (humerus) nor the hindlimb (femur) exhibits sexual dimorphism in size or in overall 2D shape.

### 3.2. Shape Variation

**Figure 1:**
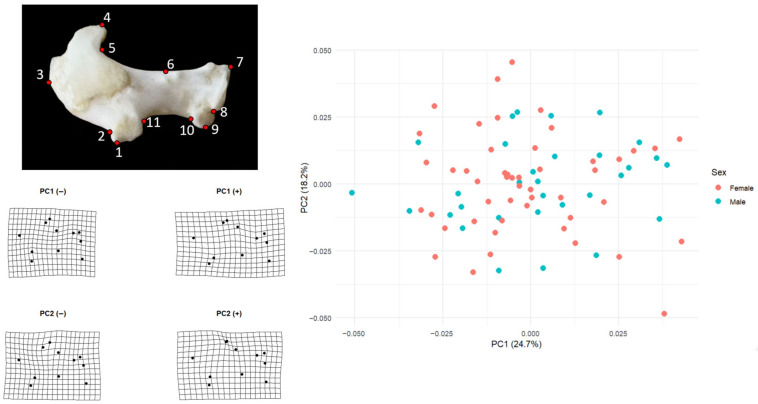
Humeral landmark configuration and major axes of shape variation. The figure illustrates the 11 two-dimensional landmarks used to describe the shape of the left humerus, together with the principal component analysis (PCA) scatterplot and the deformation grids that summarize the main directions of variation. In the PCA plot, PC1 accounts for 24.7% of total humeral shape variation and PC2 accounts for 18.2%; female specimens are shown in red and male specimens in turquoise. The substantial overlap between the two color clouds indicates that males and females occupy the same morphospace and that sex does not structure humeral shape.

Principal component analysis of the left humerus showed that the first two components captured a moderate proportion of total shape variation (PC1 = 24.7%, PC2 = 18.2%) (Figure 1). Female scores on PC1 (mean = 0.00077, SD = 0.0292, *n* = 50) and male scores (mean = −0.00120, SD = 0.0164, *n* = 32) were essentially centered on zero and overlapped broadly along the axis, indicating no sex-related displacement on the main direction of shape change. A similar pattern was visible on PC2, where females averaged −0.00196 (SD = 0.0203) and males 0.00306 (SD = 0.0189); the two groups formed a single continuous cloud in PC1–PC2 space.

For the left femur, the pattern was almost identical. PC1 explained 32.2% of the variation and PC2 16.2%. Females had mean PC1 and PC2 scores very close to zero (PC1 mean = 0.00020, SD = 0.00851; PC2 mean = 0.00062, SD = 0.00631; *n* = 50), and males showed likewise minimal displacement (PC1 mean = −0.00031, SD = 0.00805; PC2 mean = −0.00097, SD = 0.00520; *n* = 32). In both elements, male and female convex hulls would occupy the same region of morphospace, confirming that the principal axes of shape variation are shared between sexes and that sex does not structure the PCA space.

Shape changes along PC1 mainly describe a transition from a more compact humerus to a more elongated and distally expanded one. At the negative end of PC1 the distal landmarks, especially those around the condylar region (landmarks 7–10), lie closer to the shaft and the overall grid is slightly compressed. At the positive end, the same distal landmarks shift outward and the grid opens horizontally, producing a humerus in which the distal portion is positioned farther away from the proximal part. Proximal landmarks around the humeral head (landmarks 4–5) also follow this displacement, so PC1 can be interpreted as a general axis of distal–proximal elongation and lateral displacement.

Variation along PC2 captures a different aspect of shape, best described as a gentle dorsoventral bending of the element. In the negative PC2 configuration the central part of the shaft and the ventral–distal landmarks are displaced slightly downward, giving the grid a sagging appearance. In the positive configuration, the upper margin rises and the distal ventral region is drawn inward, producing the opposite curvature. PC2 therefore reflects changes in how much the humerus is arched or deflected rather than how long or laterally expanded it is. Taken together, these two axes show that most humeral shape differences in the sample concern relatively subtle shifts in the distal end and in the curvature of the shaft, rather than sex-related differences.

**Figure 2:**
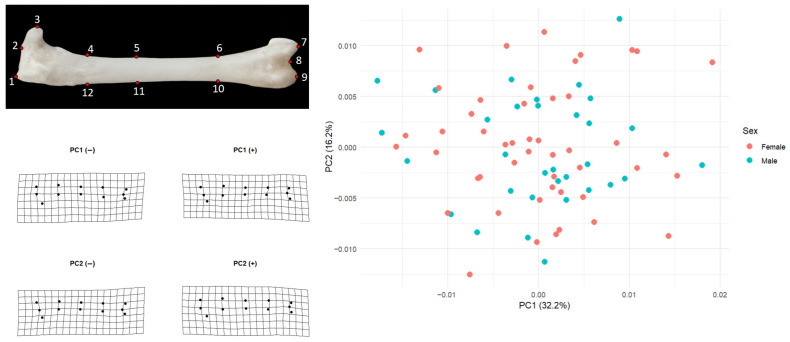
Femoral landmark configuration and major directions of shape variation. This figure presents the two-dimensional landmark configuration of the left femur defined by 12 fixed points, together with the PC1–PC2 scatterplot and the deformation grids that illustrate the principal patterns of femoral shape change. In the PCA plot, PC1 explains 32.2% of total femoral shape variation and PC2 explains 16.2%. Female individuals are shown in red and males in turquoise. The fact that both groups cluster largely within the same region indicates that femoral shape is not clearly separated by sex and that males and females occupy essentially the same morphospace.

Shape change along PC1 mainly concerns longitudinal (proximal–distal) structuring of the femur and a redistribution of mass around the distal end (Figure 2). At the negative end of PC1 the shaft appears straighter and slimmer, and the distal landmarks (especially 7–9) lie closer to the general axis of the bone; the grid looks slightly closed inward. At the positive end, the midshaft is drawn very slightly inward while the distal landmarks shift outward and anteriorly, producing a configuration in which the distal portion is more clearly separated from the shaft and the tip of the bone shows a mild lateral expansion. Thus, PC1 can be interpreted as an axis describing changes in the form of the distal extremity and in the relationship between the shaft and the distal end.

PC2 captures a subtler pattern of bending or deflection along the length of the femur. At the negative extreme, the grid sags slightly downward and the shaft landmarks are displaced ventrally, making the femur appear a bit more curved in that direction. At the positive extreme, the shaft rises dorsally, the distal end is lifted, and the element becomes straighter or even slightly dorsally arched. Consequently, PC2 reflects how much the femur is deflected upward or downward along its long axis rather than changes in overall size. Taken together, these two axes show that most femoral shape differences in the sample relate to the relative positioning of the shaft and distal end and to the degree of curvature along the bone, whereas sex does not make a marked contribution to this pattern.

### 3.3. Allometric Relationships (Size–Shape)

The regression analyses showed that size explains only a small, but detectable, portion of shape variation in these elements. For the humerus, shape regressed on log-transformed centroid size was statistically significant (R^2^ = 0.0486; F_1__,__80_ = 4.09; *p* = 0.007). This indicates that about 4.9% of the humeral shape differences among individuals can be attributed to differences in overall size. Although this is a modest effect, it is real: larger swifts tend to have a slightly altered humeral configuration compared to smaller ones. In geometric morphometric terms, this can be described as a weak but significant allometric pattern in the humerus.

In contrast, the femur showed only a marginal size effect (R^2^ = 0.0236; F_1__,__81_ = 1.96; *p* = 0.058). Here, size accounts for roughly 2–3% of shape variation, and the *p*-value is just above the conventional 0.05 threshold. This suggests that femoral shape is less constrained or less influenced by overall body size than humeral shape in this sample. Put differently, most of the femoral shape differences observed among individuals are not driven by size, but by other sources of variation (individual differences, asymmetry, or measurement noise).

### 3.4. Directional and Fluctuating Asymmetry in the Humerus and Femur

For both bones, two-way Procrustes ANOVA showed that the left–right (directional asymmetry, DA) difference was statistically significant, but its magnitude differed between elements. In the humerus, the “side” effect explained about 13.8% of total shape variance (SS = 0.04817, F = 42.00, *p* = 0.001) (Table 1). This indicates a systematic left–right shift in the humerus, i.e., most individuals deviate in the same direction. In the femur, however, the “side” effect was weaker (R^2^ = 0.054; F = 19.27; *p* = 0.001); thus, the femur also shows directional asymmetry, but to a lesser extent than the humerus. In other words, DA is most pronounced in the humerus and least pronounced in the femur.

In contrast, the largest source of variance in both bones was the “ID (individual)” term. In the humerus, the individual term accounted for 59.2% of the variance (F = 2.19, *p* = 0.001), and in the femur it accounted for 71.6% (F = 3.11, *p* = 0.001). This is expected in paired (left–right) datasets, because this term captures both overall among-individual shape differences and part of the individual-specific asymmetry (the FA component). The particularly high ID contribution in the femur suggests that the individual-level, non-directional component of asymmetry (i.e., fluctuating/asystematic asymmetry) is more prominent in the femur than in the humerus.

In summary, left–right differences are statistically significant in both bones; this directional difference is stronger in the humerus and weaker in the femur; and the largest share of asymmetry in both elements is concentrated at the individual level.

## 4. Discussion

Our results show clear evidence of directional asymmetry (DA) in the common swift, with the humerus exhibiting a stronger side effect (13.8% of total shape variance) than the femur (5.4%). DA is typically interpreted as the morphological imprint of consistent, side-biased functional demands rather than stochastic developmental noise [13,15]. This interpretation fits the biology of *Apus apus*: swifts spend an extraordinary proportion of the annual cycle on the wing and fly at high speeds with remarkably repeatable kinematics [2,7]. Thus, the forelimb is exposed to frequent, similarly oriented mechanical loads, whereas the femur is used mainly for clinging and limited support; the greater DA in the humerus therefore accords with the expectation that repetitive, directional loading fosters systematic left–right differences [25,26]. Comparable associations between intensive or asymmetric use and skeletal/functional asymmetries are well documented in humans and other mammals [27,28,29,30] and in experimental vertebrate models [31], reinforcing a functional link rather than a measurement artifact.

At the same time, the fact that the largest source of variation in both bones was the “individual/ID” term shows that a substantial portion of shape variability in swifts is concentrated at the among-individual level. In the humerus this term accounted for about 59% of the variance, and in the femur for more than 71%. This is important for two reasons. First, in this data structure, where each side was digitized only once, part of the individual-specific left–right difference (i.e., the fluctuating component) is also absorbed into this term, so it reflects not only general shape differences among individuals but also their differing propensity for asymmetry [15,25]. Second, the fact that the femur shows a higher individual component despite having lower DA suggests that, although the leg is functionally less stressed, its shape is somewhat freer to vary among individuals. The humerus shows a more constrained pattern of shape variation, which is consistent with stronger functional demands associated with flight, although other factors such as developmental or genetic constraints could also contribute to this pattern.

The results of this study indicate that small but consistent differences in humeral shape can occur in the common swift, a highly aerial species that spends most of its life in the air, without necessarily compromising aerodynamic efficiency. Thus, high overall symmetry of the wing outline may be compatible with a limited degree of skeletal directional asymmetry at the level of the humerus. Future studies could test whether similar patterns of skeletal directional asymmetry occur in other strong fliers and to what extent such asymmetries are associated with variation in soft tissues or in the external wing morphology (e.g., feather arrangement and wing outline).

Allometric effects further support a functional interpretation. Centroid size explained ~5% of humeral shape, but femoral allometry was only marginal. Size–shape associations of this magnitude are common in geometric morphometrics [32,33], and their clearer expression in the humerus suggests that the functionally constrained, flight-critical element is also more size-sensitive. Together with the higher DA, this points to stronger developmental canalization of humeral form around flight demands, whereas the femur, under weaker and less directional loads, appears freer to vary among individuals.

Finally, the almost complete overlap of males and females in PCA space, together with the non-significant Procrustes tests for sex, shows that the asymmetry pattern we detected is not driven by sexual dimorphism. This supports the view that the source of directional asymmetry is not sex or overall sexual size differences but rather side-biased loading and the species’ aerial lifestyle. A limitation of the present study is that each side was digitized only once, so FA could not be tested as a separate “individual × side” term; however, the residual component accounted for about 27% of total shape variance in the humerus and 23% in the femur, suggesting that even with replicated data the humerus would likely show slightly higher within-individual asymmetry. Taken together, these findings indicate that, in this extremely aerial bird, forelimb and hindlimb bones do not have the same level of symmetry, and that the more heavily used forelimb element tends to show stronger directional asymmetry than the hindlimb, consistent with, but not proving, a link between habitual use and asymmetric shape change.

In this study we used a single-digitizing protocol, because our primary objective was to detect and compare directional asymmetry between two functionally different elements rather than to fully partition fluctuating asymmetry. In a Procrustes ANOVA framework, a strong and significant ‘side’ effect—such as the 13.8% we observed in the humerus—indicates that left–right differences are systematic and cannot be explained by random digitizing noise [15,25]. In other words, if measurement error were dominant, the side term would not emerge as clearly as it did here. At the same time, the residual component (≈27% in the humerus and ≈23% in the femur) shows that some within-individual variation was still captured even without replicated landmarking. For testing a DA-focused hypothesis, namely that the more intensively used wing element departs more from bilateral symmetry than the hindlimb, this level of precision is sufficient. A full design with repeated measurements and an explicit individual × side term would mainly have refined the FA estimate but would not have changed the central conclusion that DA is present and is stronger in the humerus.

A major limitation of the present study is that no direct ecological or physiological covariates (e.g., migration distance, body condition, age class, or health status) were available for the individuals examined, nor was precise chronological age known for each specimen. Although all individuals were fully ossified and showed no macroscopic signs of immaturity, they may nevertheless represent a range of adult ages, so some of the observed shape variation could reflect residual age-related differences that we were unable to quantify. As a result, we could not test whether the relatively large among-individual component of shape variation was actually linked to differences in environmental exposure or workload, which are often invoked in studies of developmental instability and asymmetry. In addition, the analysis was restricted to only two long bones, the humerus and the femur, so the inference that “the more flight-related element shows stronger directional asymmetry” is, at this stage, bone-specific. Our sample was also modest, which means that extending the approach to more skeletal elements, especially more distal and morphologically complex ones (ulna, carpometacarpus, tarsometatarsus), will require a denser and more carefully standardized digitizing workflow to keep measurement noise low. Future work that combines bone-level asymmetry with individual ecological data and a broader set of limb elements would allow a more robust evaluation of how functional loading translates into skeletal asymmetry in highly aerial birds.

## 5. Conclusions

This study showed that in the common swift (*Apus apus*), a species with an extremely aerial lifestyle, skeletal symmetry is not expressed at the same level across all elements. The humerus, which is directly involved in flight, exhibited higher directional asymmetry (DA) than the femur, which is used more limitedly in this species, indicating that the forelimb is more strongly shaped in this bird. The fact that the individual (ID) term explained a large proportion of variance in both the humerus and the femur suggests that there are marked among-individual differences in overall bone shape and that these differences are also reflected in asymmetry levels. The slight allometric effect observed in the humerus implies that the flight-related element may be more sensitive to size, whereas the weaker relationship in the femur indicates that the shape of the hindlimb is able to vary more freely. Overall, our findings indicate that in highly aerial birds the symmetry levels of forelimb and hindlimb bones are not the same, and that forelimb bones display the traces of asymmetry more clearly. Future studies that incorporate ecological/physiological variables at the individual level and include more distal elements will be useful for testing how far this pattern can be generalized to the avian skeleton as a whole.

## Figures and Tables

**Table 1:** Procrustes ANOVA results for bilateral shape variation in the humerus and femur, showing contributions of directional asymmetry (side), individual effects, and residual (FA + error) components.

Bone	Effect	df	SS	R^2^	F	*p*
Humerus	side (DA)	1	0.04817	0.138	42.00	0.001
Humerus	ID (individual)	82	0.20606	0.592	2.19	0.001
Humerus	Residual (FA + error)	82	0.09404	0.270	–	–
Femur	side (DA)	1	0.001953	0.054	19.27	0.001
Femur	ID (individual)	82	0.025813	0.716	3.11	0.001
Femur	Residual (FA + error)	82	0.008308	0.230	–	–

## Data Availability

The data presented in this study are available upon request from the corresponding author (O.G.).
